# Invasive Gastric Adenocarcinoma Arising in a Giant Pedunculated Polyp Causing Intermittent Gastric Outlet Obstruction: A Case Report

**DOI:** 10.7759/cureus.116294

**Published:** 2026-09-15

**Authors:** Btissam Essadi, Ayoub Bouziane, Wiam Rabhi, Chaimae Bekhakh, Anass Haloui, Amal Bennani, Ouiam Elmqaddam, Hajar Koulali, Ghizlane Kharrasse, Zahi Ismaili, Abdelkrim Zazour

**Affiliations:** 1 Faculty of Medicine and Pharmacy, Department of Hepato-Gastroenterology, Mohammed VI University Hospital Center, Mohammed First University, Oujda, MAR; 2 LAPOD (Research Laboratory of Pathology and Digestive Oncology) Faculty of Medicine and Pharmacy, Mohammed First University, Oujda, MAR; 3 Mohammed VI University Hospital Center, Mohammed First University, Faculty of Medicine and Pharmacy, Department of Hepato-Gastroenterology, Oujda, MAR; 4 Faculty of Medicine and Pharmacy, Department of Pathology, Mohammed VI University Hospital, Mohammed First University, Oujda, MAR; 5 Faculty of Medicine, Department of Pathology, Mohammed VI University Hospital, Mohammed First University, Oujda, MAR

**Keywords:** endoscopic resection, gastric adenocarcinoma, gastric adenoma, gastric outlet obstruction, gastric polyp, pyloric prolapse

## Abstract

A 69-year-old woman with no significant medical or surgical history presented with a three-month history of epigastric pain, early postprandial vomiting, and dyspepsia. Thoracoabdominopelvic computed tomography showed marked esophageal and gastric stasis upstream of an endoluminal polypoid mass extending toward the duodenal bulb. Upper gastrointestinal endoscopy revealed an approximately 4-cm pedunculated polyp arising from the gastric body and intermittently prolapsing through the pylorus. Initial superficial biopsies showed inflammatory and ulcerative changes without evidence of malignancy. Endoscopic snare polypectomy was subsequently performed. Histopathological examination of the resected specimen revealed a moderately differentiated adenocarcinoma arising in a tubular adenoma containing both low- and high-grade dysplasia. The tumor invaded the submucosa to a depth of 2,400 µm, without lymphovascular invasion, perineural invasion, or tumor budding. Because the depth of submucosal invasion exceeded the criteria for curative endoscopic resection, subtotal gastrectomy with lymph node dissection was performed. No residual tumor was identified, and all 13 examined lymph nodes were negative for metastasis.

This case illustrates a rare presentation of invasive gastric adenocarcinoma arising in a large pedunculated gastric adenoma and causing intermittent gastric outlet obstruction through pyloric prolapse. It emphasizes the limitations of superficial biopsies in suspicious polypoid lesions and the importance of complete histological assessment and multidisciplinary management after non-curative endoscopic resection.

## Introduction

Gastric polyps are a heterogeneous group of mucosal lesions that protrude into the gastric lumen [1-3]. They are most often discovered incidentally during upper gastrointestinal endoscopy performed for dyspepsia, anemia, gastrointestinal bleeding, or other clinical indications [2]. Their management depends primarily on the histological type, size, endoscopic appearance, presence of dysplasia, and characteristics of the surrounding gastric mucosa [1,2].

The main epithelial types include fundic gland polyps, hyperplastic polyps, and gastric adenomas. Gastric adenomas are of particular clinical importance because they are true premalignant lesions [1,2]. According to the World Health Organization classification, gastric adenomas encompass four major histologic subtypes: intestinal-type adenoma, foveolar-type adenoma, pyloric gland adenoma, and oxyntic gland adenoma [4]. These subtypes differ in their clinicopathologic characteristics and malignant potential. Intestinal-type adenomas are the most common and typically arise in a background of chronic atrophic gastritis and intestinal metaplasia, often associated with Helicobacter pylori infection or autoimmune gastritis; among gastric adenomas, they carry the highest risk of progression to high-grade dysplasia and adenocarcinoma. Sporadic foveolar-type adenomas generally have a lower malignant potential, whereas pyloric gland adenomas are recognized precursor lesions with a well-documented risk of high-grade dysplasia and associated adenocarcinoma. Oxyntic gland adenomas usually exhibit low-grade cytologic atypia and a relatively indolent clinical course, although rare progression toward fundic gland-type adenocarcinoma may occur [4]. Overall, the risk of malignant transformation is influenced not only by histologic subtype but also by lesion size, high-grade dysplasia, villous architecture, and suspicious endoscopic features [1,2,4,5].

Gastric polyps are usually asymptomatic; however, large pedunculated lesions may rarely prolapse through the pylorus and cause intermittent gastric outlet obstruction [1,2].

We report the case of a 69-year-old woman with a moderately differentiated invasive gastric adenocarcinoma arising in a large pedunculated tubular adenoma of the gastric body, revealed by intermittent gastric outlet obstruction secondary to pyloric prolapse. This case highlights the limitations of superficial biopsies in suspicious polypoid lesions, the diagnostic value of complete endoscopic resection, and the importance of multidisciplinary management after non-curative endoscopic resection.

## Case presentation

Medical history and clinical presentation

A 69-year-old Moroccan woman with no significant medical or surgical history, no history of alcohol consumption or tobacco use, and no family history of malignancy was admitted with symptoms suggestive of gastric outlet obstruction, including severe epigastric pain, early postprandial vomiting of food contents, and dyspepsia. The symptoms had been evolving for three months, without deterioration in her general condition.

Physical examination

On admission, the patient was in good general condition, with a World Health Organization performance status of 0, a body mass index of 22 kg/m^2^, and a Nutritional Risk Index of 106. Abdominal examination revealed a soft, non-tender abdomen, without guarding, palpable mass, or hepatosplenomegaly.

Laboratory findings

Laboratory investigations showed no anemia or significant inflammatory response. Hemoglobin was 12.6 g/dL, the platelet count was 227,000/mm^3^, the white blood cell count was 4,450/mm^3^, prothrombin time was 100%, and C-reactive protein was 1.6 mg/L. Renal and liver function tests were within normal limits, and serum albumin was 43 g/L. The carcinoembryonic antigen level was normal at 2.7 ng/mL. Iron deficiency without anemia and severe vitamin D deficiency were noted, with a ferritin level of 10 ng/mL and a serum vitamin D level of 8.7 ng/mL.

Imaging findings

**Figure 1:**
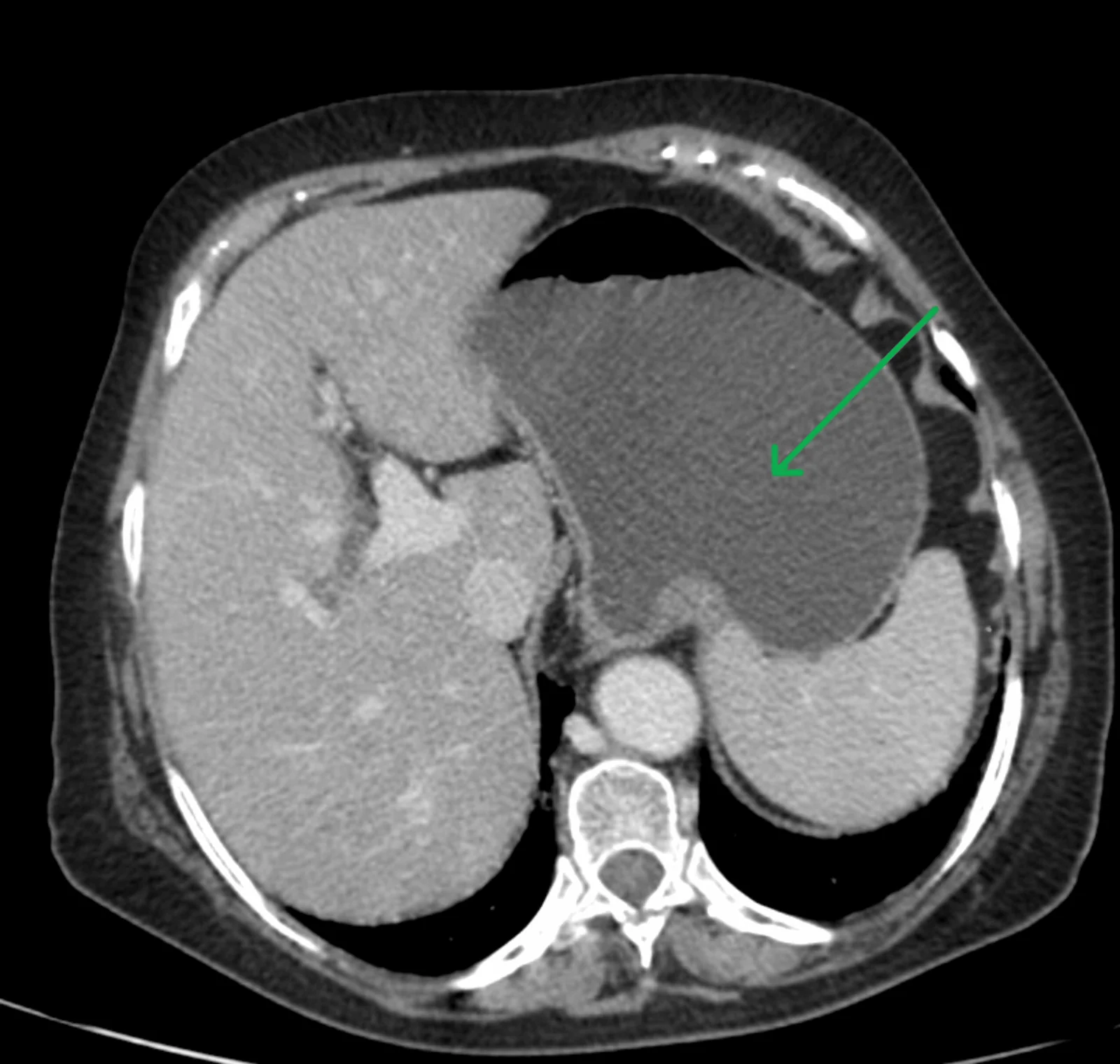
Contrast-enhanced thoracoabdominopelvic computed tomography showing marked gastric dilatation and stasis (green arrow).

**Figure 2:**
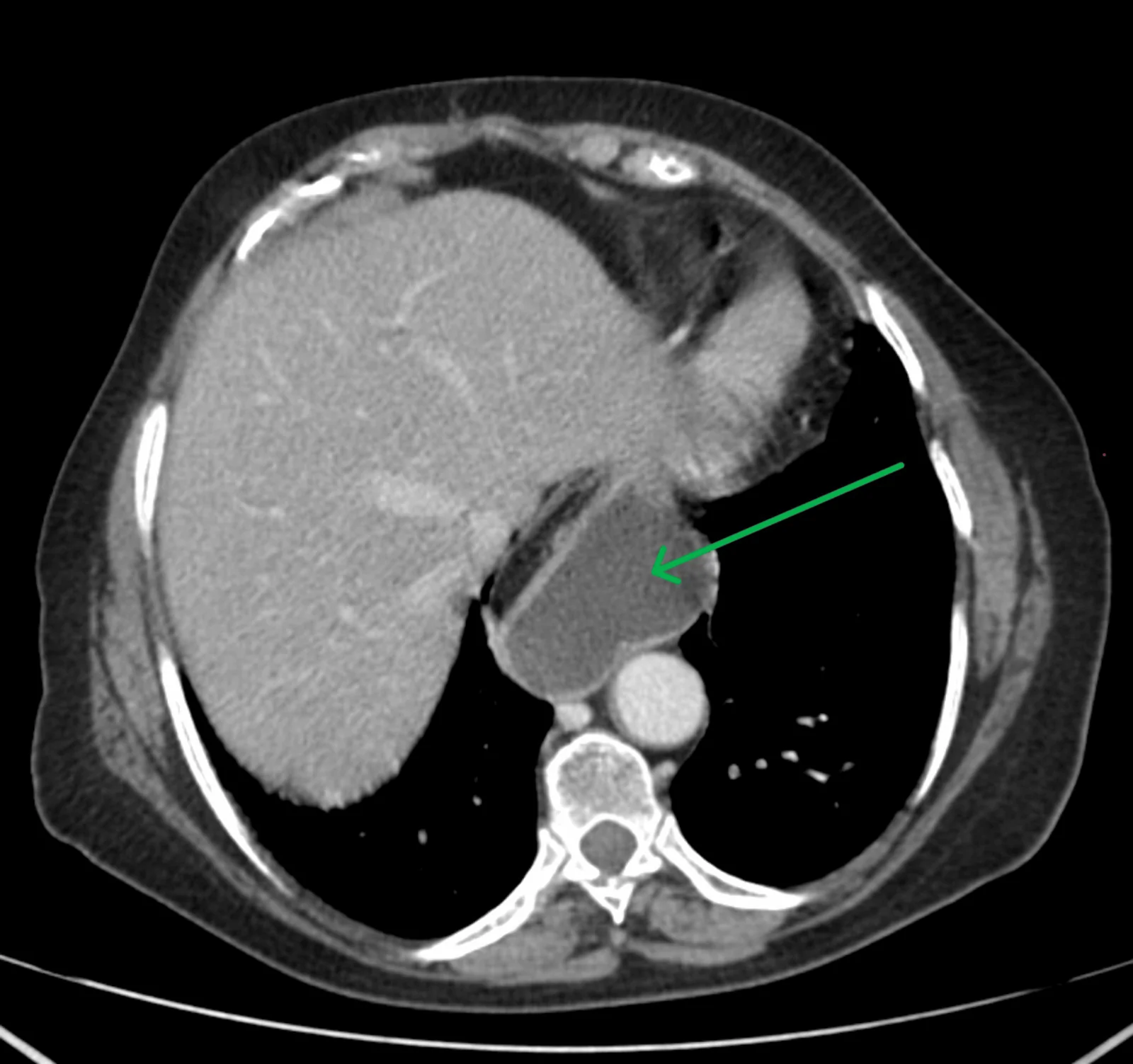
Contrast-enhanced thoracoabdominopelvic computed tomography showing esophageal dilatation (green arrow).

**Figure 3:**
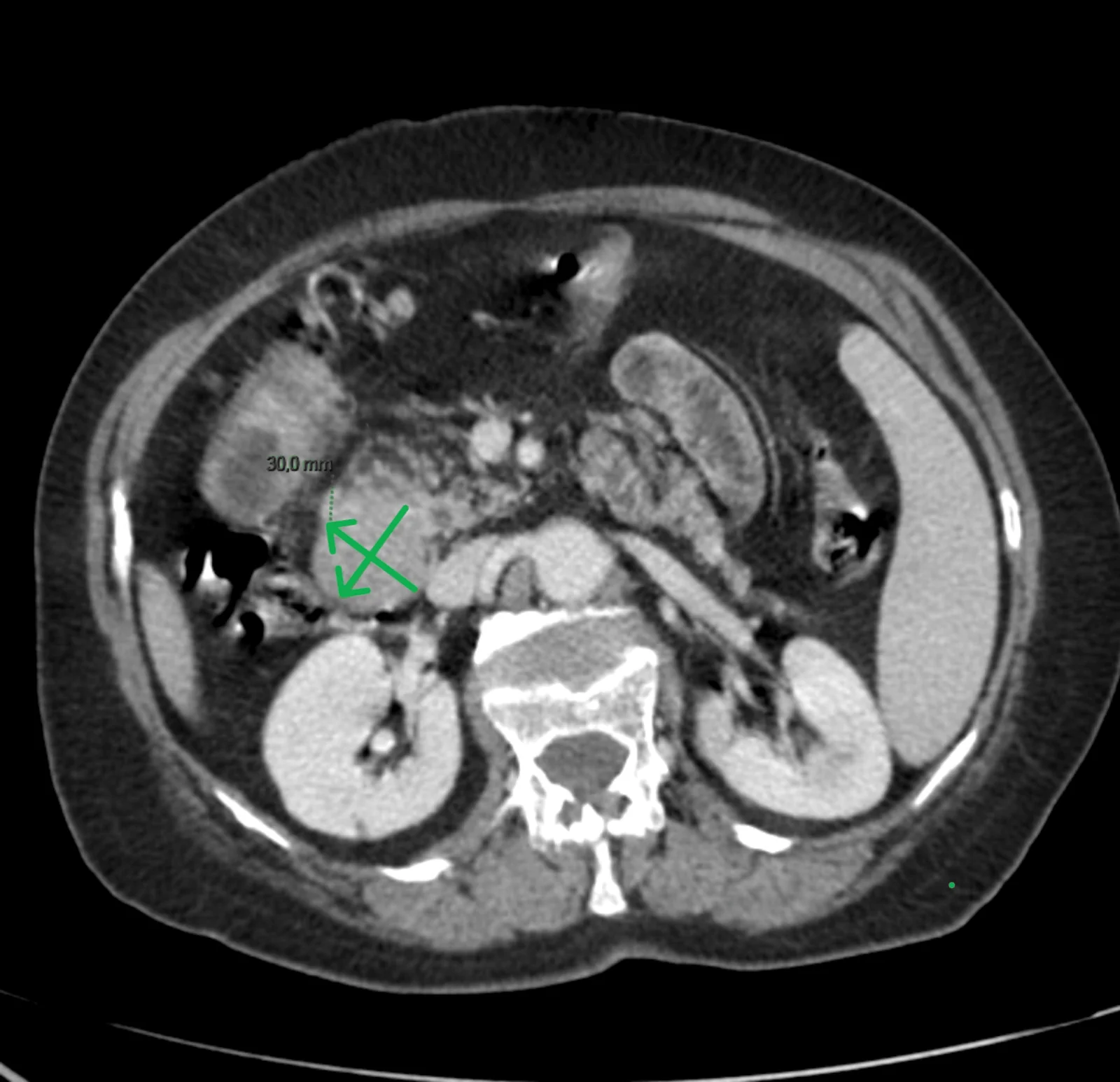
Contrast-enhanced abdominal computed tomography, axial view, showing an endoluminal polypoid soft-tissue mass measuring 30 mm (green arrows).

**Figure 4:**
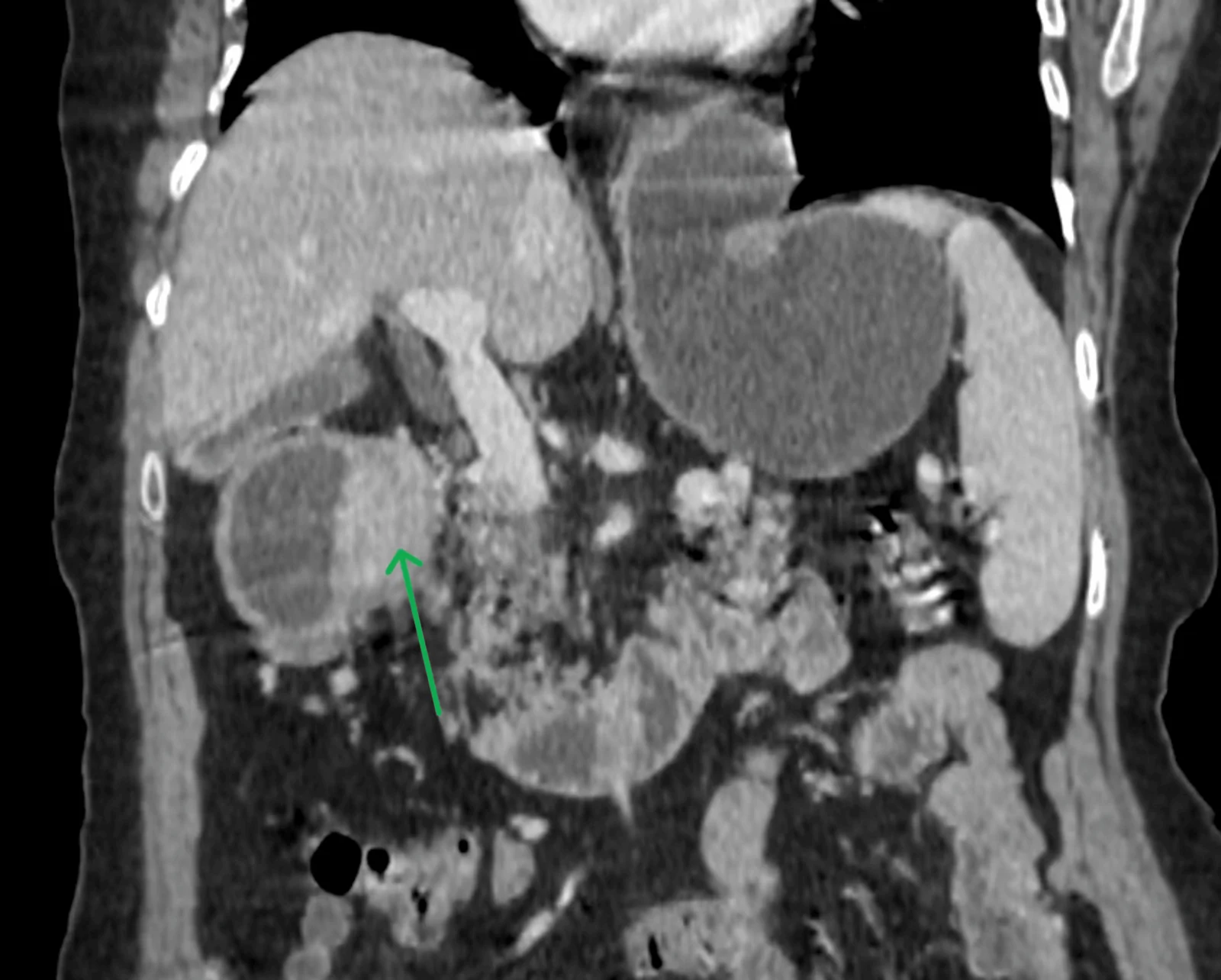
Contrast-enhanced abdominal computed tomography, coronal reconstruction, showing extension of the polypoid lesion toward the duodenal bulb (green arrow).

Thoracoabdominopelvic computed tomography revealed marked esophageal and gastric dilatation with stasis (Figures 1, 2), upstream of an endoluminal polypoid soft-tissue mass extending from the pyloric region toward the duodenal bulb and reaching the second portion of the duodenum. The lesion measured 32 × 30 mm, with an estimated craniocaudal length of 65 mm. No pathologically enlarged lymph nodes or distant metastases were identified (Figures 3, 4).

Diagnostic upper gastrointestinal endoscopy

**Figure 5:**
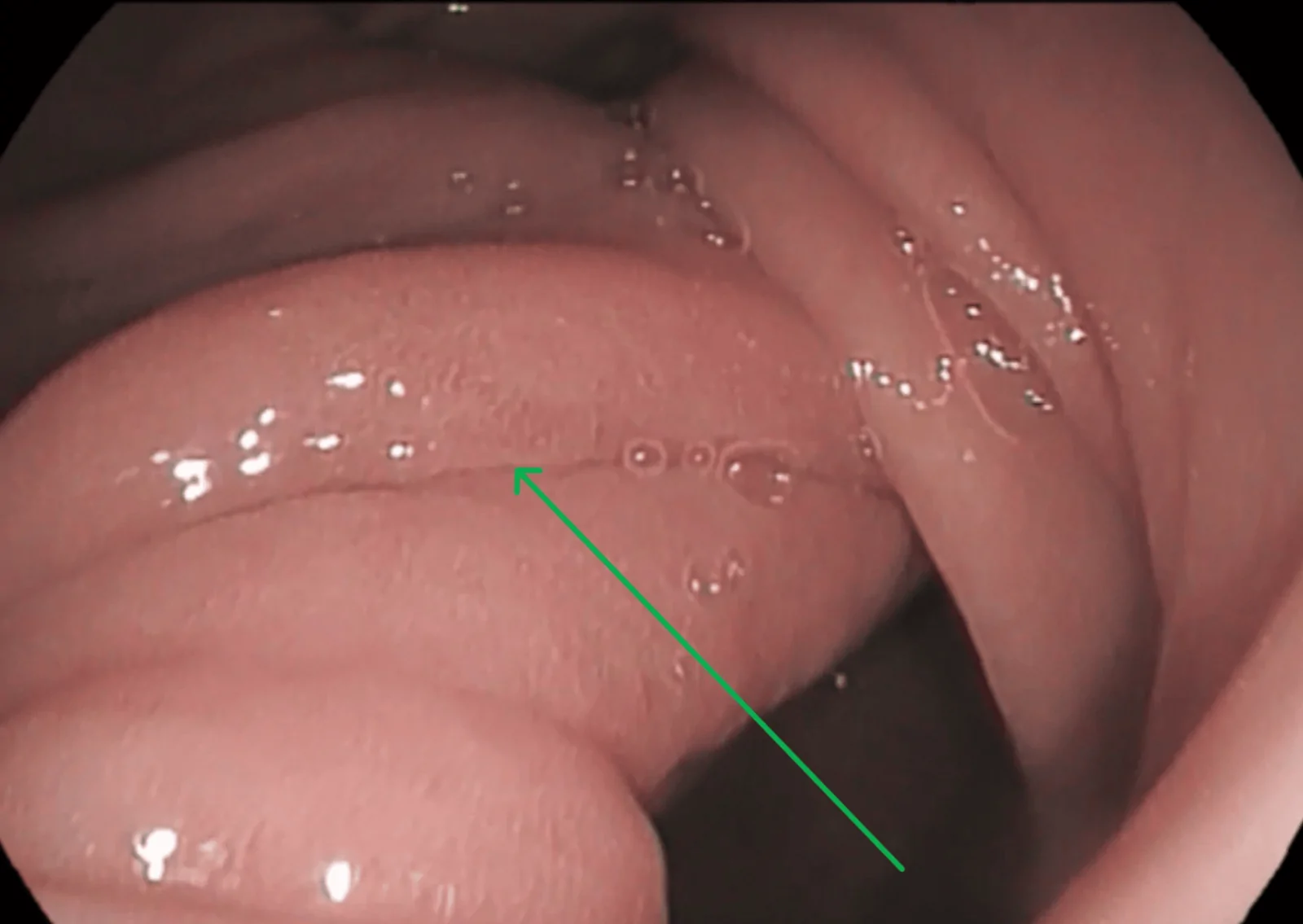
Endoscopic image showing the broad stalk of the polyp prolapsing through the pylorus (green arrow).

Upper gastrointestinal endoscopy revealed a large, broad-based pedunculated polyp with a raspberry-like surface, measuring approximately 4 cm and arising from the gastric body. The lesion intermittently prolapsed through the pylorus into the duodenal bulb and returned to the gastric cavity during withdrawal of the endoscope (Figure 5).

A small adjacent sessile polyp, approximately 1 mm in diameter and with a hyperplastic appearance, was also identified, together with another sessile polyp in the subcardial region. The antral mucosa appeared erythematous and whitish. Multiple biopsies were obtained.

Initial histopathological findings

Histopathological examination of the initial biopsy specimens obtained from the apical ulcerated area of the polyp showed acute inflammatory and ulcerative changes, with no evidence of dysplasia or malignancy in 8 of the 21 tissue specimens examined. Examination of the background gastric mucosa revealed mild chronic antral and fundic gastritis with mild atrophic changes, associated with focal complete intestinal metaplasia without dysplasia. Helicobacter pylori was not identified.

Therapeutic endoscopy

**Figure 6:**
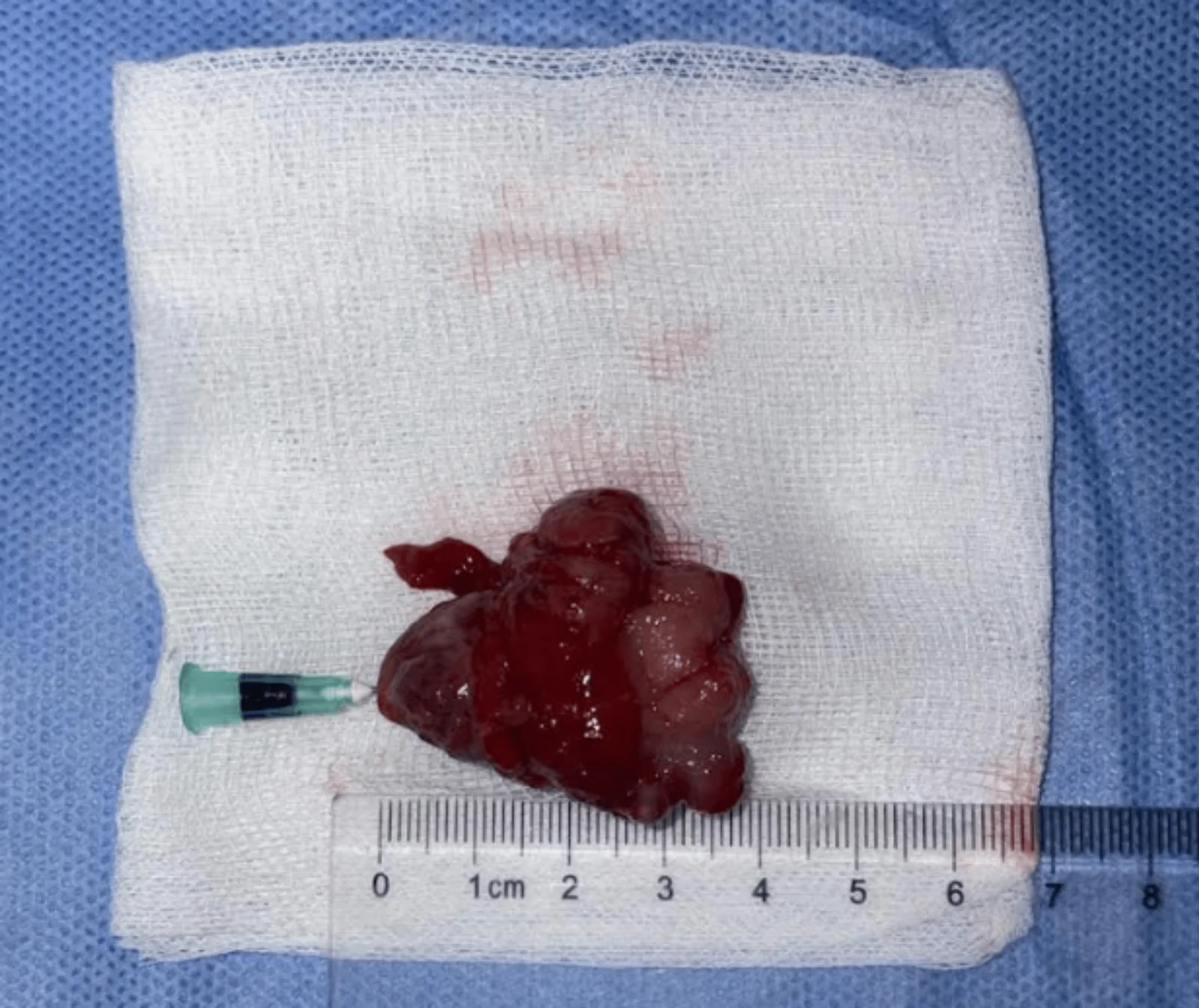
Gross appearance of the resected specimen showing a large pedunculated gastric polyp measuring approximately 4 cm.

A second upper gastrointestinal endoscopy was performed for therapeutic purposes. The large pedunculated gastric polyp was resected en bloc using a diathermic snare, after the prophylactic placement of three hemostatic clips at the base of the stalk. The resected specimen was retrieved using a Dormia basket. The resection site was subsequently closed with two additional hemostatic clips. The associated sessile polyps located in the subcardial region and gastric body were also resected. After extraction, macroscopic examination of the polypectomy specimen revealed a large pedunculated polyp measuring approximately 4 cm in its greatest dimension (Figure 6).

Histopathological examination of the polypectomy specimen

**Figure 7:**
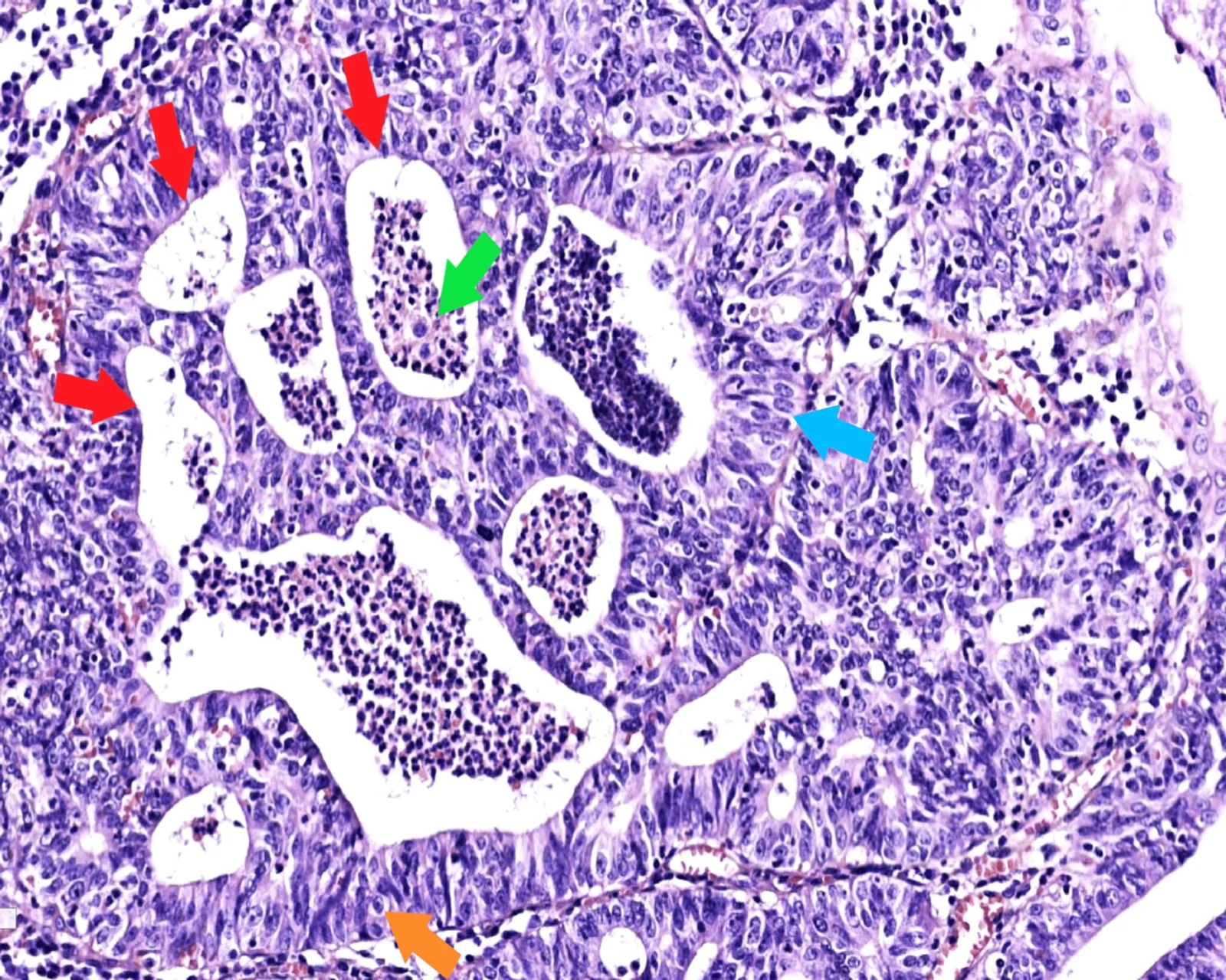
High-power histological view showing high-grade dysplasia within the adenomatous component, characterized by complex glandular crowding and cribriform architecture (red arrows), intraluminal necrosis (green arrow), loss of cell polarity (blue arrow), and marked nuclear atypia with nuclear enlargement and prominent nucleoli (orange arrow). Hematoxylin, eosin, and saffron staining, ×20.

**Figure 8:**
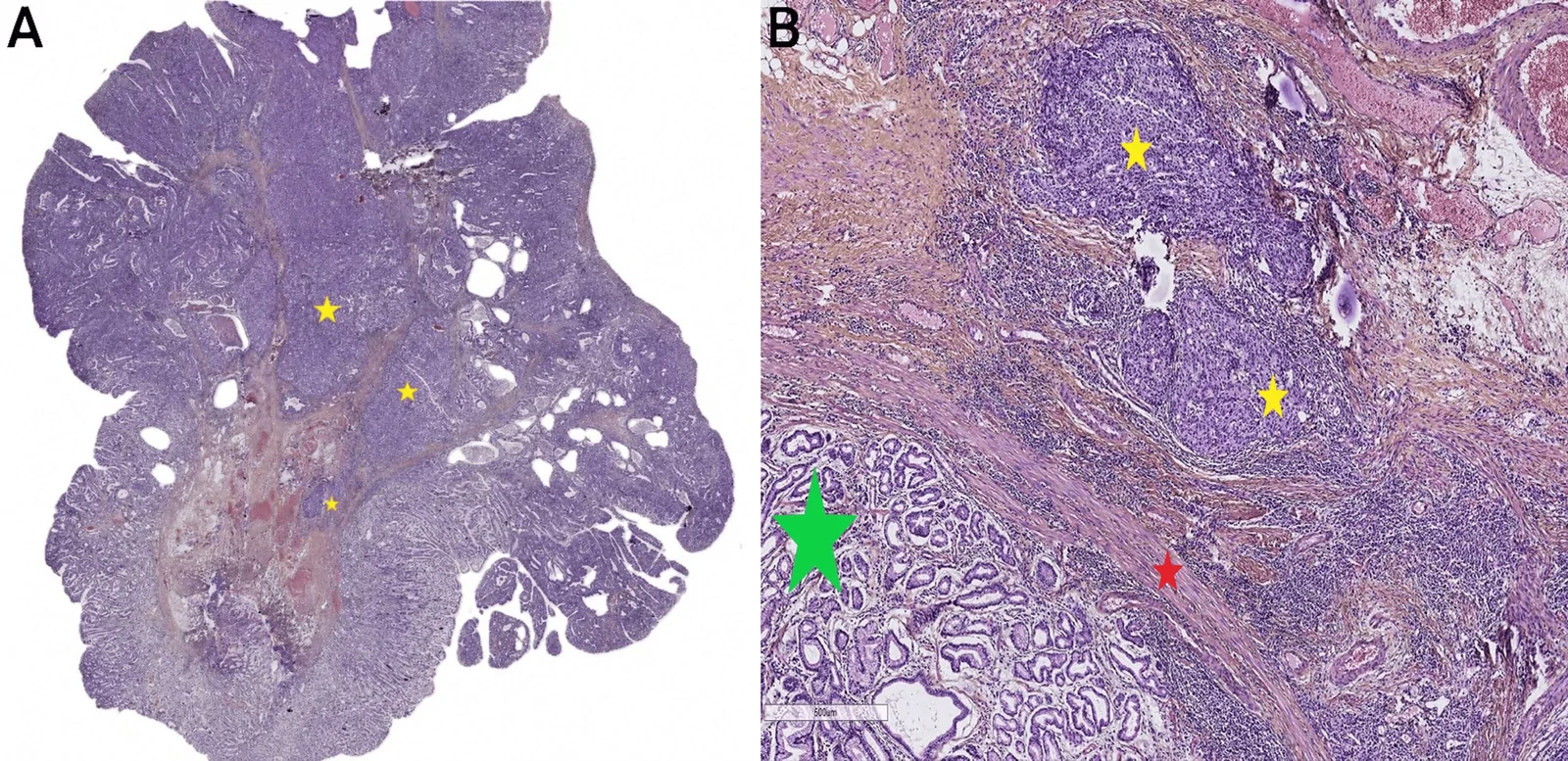
Histological demonstration of submucosal invasion. (A) Low-power view of the pedunculated tubular adenoma showing foci of moderately differentiated invasive adenocarcinoma within the head and stalk of the polyp (yellow asterisks), ×2.5. (B) Higher-power view showing invasive adenocarcinoma within the submucosa (yellow stars), beneath the muscularis mucosae (red star), with preservation of the overlying gastric mucosa (green star), ×5. Hematoxylin, eosin, and saffron staining.

**Figure 9:**
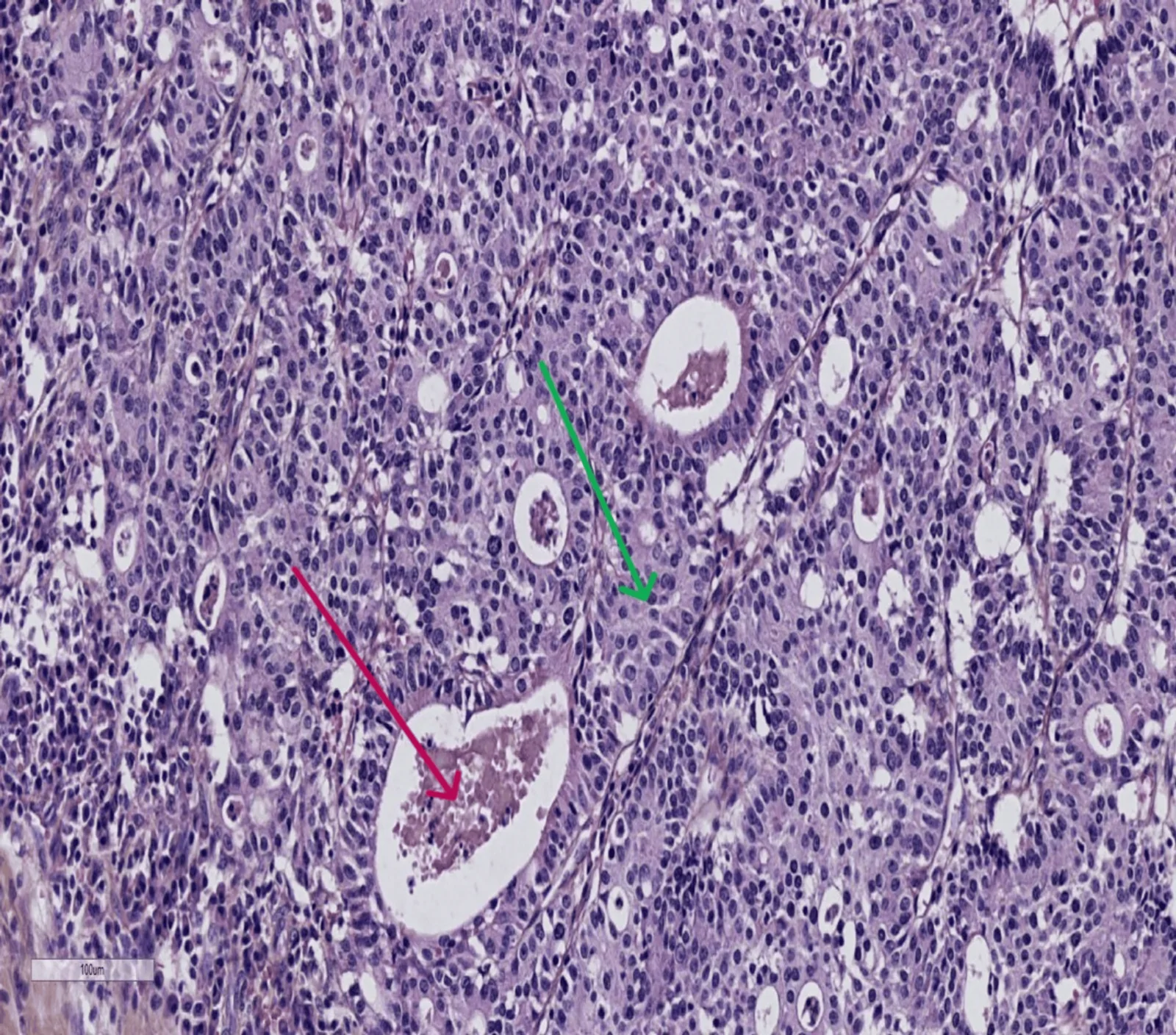
High-power histological view of a moderately differentiated invasive adenocarcinoma showing cribriform architecture (green arrow) and foci of intraluminal necrosis (red arrow). Hematoxylin, eosin, and saffron staining, ×20.

Histopathological examination of the polypectomy specimen revealed a tubular adenoma containing low-grade dysplasia in approximately 20% of the lesion and high-grade dysplasia in approximately 80% (Figure 7), with an associated moderately differentiated invasive adenocarcinoma (Figures 8-9).

**Figure 10:**
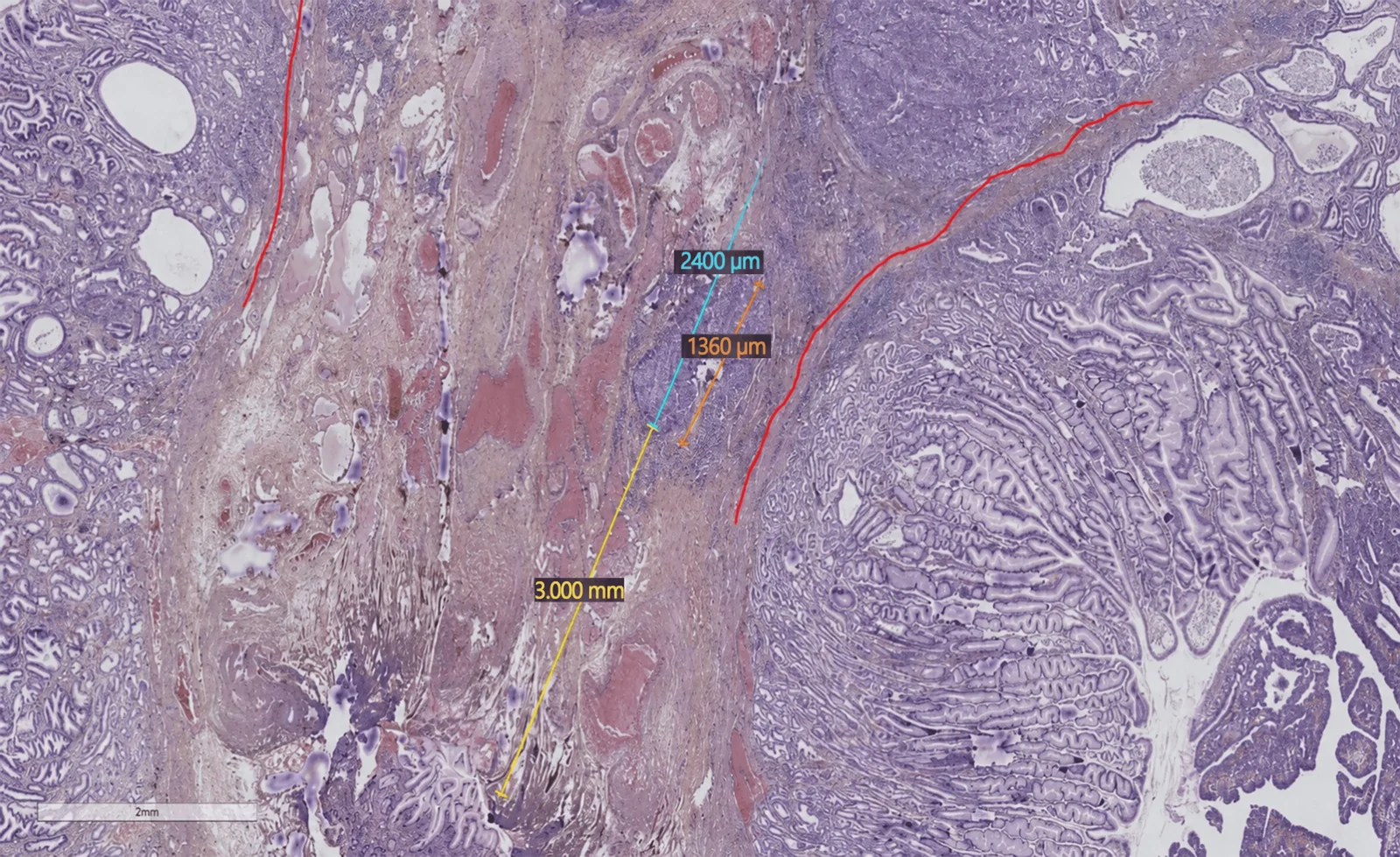
Low-power view showing a maximal invasion depth of 2,400 µm (blue line) and a minimum distance of 3,000 µm (3 mm) between the invasive carcinoma and the deep resection margin (yellow line). The invasive focus within the polyp stalk measures 1,360 µm in greatest dimension (orange line).

**Figure 11:**
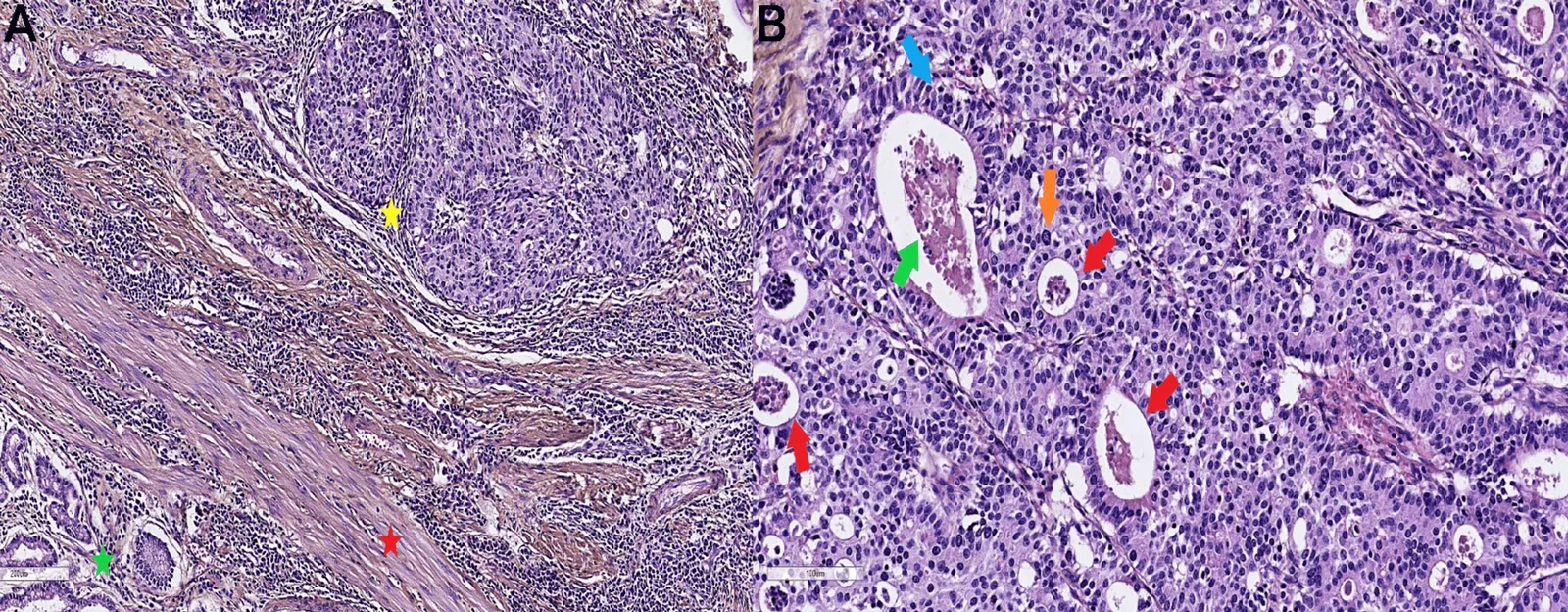
Higher-magnification histological assessment of the invasive component. (A) Intermediate-power view demonstrating invasive adenocarcinoma within the submucosa (yellow star), beneath the muscularis mucosae (red star), with the overlying normal gastric mucosa indicated by the green star, ×10. (B) Higher-power view of the invasive adenocarcinoma showing cribriform glandular architecture (red arrows), marked cytological atypia and anisokaryosis (orange arrow), loss of nuclear polarity (blue arrow), and intraluminal necrosis (green arrow), ×20. Hematoxylin, eosin, and saffron staining.

The carcinoma extended through the muscularis mucosae into the submucosa, with a maximum depth of invasion of 2,400 μm. The invasive focus within the polyp stalk measured 1,360 μm in greatest dimension. The deep resection margin was free of tumor, with a minimum clearance of 3 mm from the invasive carcinoma (Figures 10-11). These findings, including the measurements of invasion depth, were confirmed on a second histopathological review.

No lymphatic or vascular invasion, perineural invasion, or tumor budding was identified. Immunohistochemical staining showed retained nuclear expression of MutL homolog 1 (MLH1), postmeiotic segregation increased 2 (PMS2), MutS homolog 2 (MSH2), and MutS homolog 6 (MSH6), consistent with a mismatch repair-proficient phenotype (pMMR).

Multidisciplinary assessment and surgical management

Given the deep submucosal invasion of 2,400 μm, which exceeded the criteria for curative endoscopic resection, the endoscopic treatment was considered non-curative despite clear resection margins and the absence of lymphovascular invasion.

The case was discussed at a multidisciplinary tumor board. Based on the histopathological examination of the polypectomy specimen and the absence of radiologically detectable lymph node or distant metastases, the tumor was classified as pT1b (sm2) cN0 cM0. Additional surgical resection with regional lymph node dissection was recommended because of the risk of occult lymph node metastasis.

The patient underwent a subtotal four-fifths gastrectomy with regional lymph node dissection and a transmesocolic gastrojejunal anastomosis.

Histopathological examination of the gastrectomy specimen revealed mild follicular, mildly active, non-atrophic chronic gastritis, with areas of complete intestinal metaplasia, without dysplasia or Helicobacter pylori infection. The proximal and distal surgical margins were free of tumor. No residual carcinoma was identified.

All 13 examined lymph nodes were negative for metastatic involvement, resulting in a final pathological stage of pT1bN0 (0/13), with no clinical or radiological evidence of distant metastasis.

The postoperative course was uneventful. Following multidisciplinary discussion, endoscopic surveillance of the gastric remnant, combined with radiological follow-up, was recommended. At the two-month postoperative follow-up visit, the patient reported dyspeptic symptoms and a 10-kg weight loss since surgery. Laboratory investigations showed a hemoglobin level of 12.2 g/dL, a platelet count of 173,000/mm^3^, a white blood cell count of 4,020/mm^3^, a serum albumin level of 44 g/L, a ferritin level of 10 ng/mL, a folate level of 6 ng/mL, a vitamin B12 level of 155 pg/mL, and a vitamin D level of 16 ng/mL. Vitamin B12 and vitamin D supplementation were initiated.

## Discussion

Gastric polyps comprise a heterogeneous group of epithelial lesions that protrude into the gastric lumen. Their reported prevalence varies according to the population studied, the indication for endoscopy, and the definitions and histological classifications used. Most gastric polyps are detected incidentally during upper gastrointestinal endoscopy, although some may cause symptoms because of their size, location, surface ulceration, or associated neoplastic transformation [1-3].

The main epithelial types include fundic gland polyps, hyperplastic polyps, and gastric adenomas. These lesions differ substantially in their pathogenesis, associated abnormalities of the surrounding gastric mucosa, malignant potential, and recommended management. A systematic endoscopic examination should therefore assess not only the polyp itself but also the background gastric mucosa, with targeted biopsies and additional topographic biopsies when atrophy, intestinal metaplasia, or other mucosal abnormalities are suspected [1-3].

Fundic gland polyps are among the most frequently encountered gastric epithelial polyps. They are mainly located in the gastric body and fundus, are generally small and multiple, and occur more frequently in middle-aged women. Most are sporadic and may be associated with prolonged proton pump inhibitor use. A minority occur in the setting of a hereditary polyposis syndrome, particularly familial adenomatous polyposis [1-3].

The malignant potential of sporadic, non-syndromic fundic gland polyps is very low. Small polyps with a typical endoscopic appearance generally do not require complete resection or systematic surveillance. Nevertheless, biopsy, resection, or further investigation should be considered when the lesions are large, ulcerated, irregular, located in the antrum, unusually numerous, or associated with dysplasia or a clinical context suggestive of a hereditary polyposis syndrome [1-3].

Hyperplastic polyps are another common epithelial type and are preferentially located in the antrum. They usually arise in a background of chronic gastric mucosal injury, including H. pylori-associated gastritis, autoimmune gastritis, gastric atrophy, intestinal metaplasia, or mucosal changes following gastric surgery. Although most hyperplastic polyps are benign, dysplasia or adenocarcinoma may occasionally develop within them [1-3].

The reported frequency of neoplastic transformation varies among published series. In a retrospective study of 274 resected hyperplastic polyps, neoplastic transformation was identified in 5.5%, including low-grade dysplasia, high-grade dysplasia, and adenocarcinoma. Transformation was significantly associated with a size greater than 1 cm, a pedunculated morphology, a history of gastrectomy, and synchronous gastric neoplasia [5]. In another cohort of 195 patients, neoplastic transformation was found in 5.1% of lesions and was associated with a size greater than 25 mm and with intestinal metaplasia or dysplasia in the adjacent gastric mucosa [6].

Accordingly, the risk of neoplastic transformation appears to increase principally with lesion size and with abnormalities in the surrounding gastric mucosa. A pedunculated morphology has also been associated with neoplastic transformation in some series, but erosion, hyperemia, or an irregular surface have not been consistently identified as independent predictors [5,6].

The management of hyperplastic polyps should include evaluation of both the lesion and the surrounding gastric mucosa. All patients with hyperplastic or adenomatous gastric polyps should be tested for H. pylori and treated when infection is present. Endoscopic resection should be considered for large, symptomatic, pedunculated, ulcerated, dysplastic, or otherwise atypical lesions, particularly when complete histological assessment is required to exclude neoplastic transformation [1-6].

Gastric adenomas are less frequent but have substantially greater clinical significance because they are true premalignant epithelial neoplasms. They are usually diagnosed in older patients, occur most frequently in the antrum, and commonly arise in a background of chronic atrophic gastritis and intestinal metaplasia [1-3,7].

Gastric adenomas encompass several histologic subtypes with distinct clinicopathologic features and malignant potential, including intestinal-type, foveolar-type, pyloric gland, and oxyntic gland adenomas [4]. Intestinal-type adenomas are the most common and carry the highest risk of malignant progression. In contrast, sporadic foveolar-type adenomas generally have a lower malignant potential, with high-grade dysplasia and carcinoma being uncommon. Pyloric gland adenomas also represent clinically important precursor lesions, with high-grade dysplasia reported in up to approximately 42% of cases and associated adenocarcinoma in approximately 12-30%. Oxyntic gland adenomas generally exhibit low-grade cytologic atypia and an indolent clinical course, with true submucosal invasion being uncommon [4].

The risk of progression from gastric adenoma to carcinoma is influenced not only by histologic subtype but also by lesion size, grade of dysplasia, depressed morphology, and suspicious endoscopic features [4,7]. In a long-term follow-up study of gastric intestinal-type adenomas, the estimated five-year rate of progression to carcinoma was 34%. A lesion size of at least 15 mm and a depressed morphology were independent predictors of malignant progression. However, this estimate was obtained from a selected cohort managed by endoscopic observation and should not be extrapolated indiscriminately to all gastric adenomas [7]. These findings emphasize the importance of accurate histopathologic characterization, particularly in large lesions or those containing high-grade dysplasia, as illustrated by the present case, in which a large tubular adenoma contained extensive high-grade dysplasia associated with invasive adenocarcinoma.

Gastric adenomas should therefore undergo complete endoscopic resection whenever technically feasible. Examination of the remaining gastric mucosa is essential to detect atrophy, intestinal metaplasia, H. pylori infection, synchronous neoplasia, and other markers of an increased risk of metachronous gastric cancer. Endoscopic surveillance after resection should be individualized according to the final histological diagnosis, completeness of resection, and condition of the background gastric mucosa [1-4,7].

Most gastric polyps remain asymptomatic. When symptoms occur, they may include epigastric pain, dyspepsia, iron-deficiency anemia, overt gastrointestinal bleeding, early satiety, nausea, and vomiting. Large pedunculated polyps arising in the antrum, prepyloric region, or distal gastric body may intermittently prolapse through the pylorus and into the duodenal bulb. This may produce a ball-valve mechanism, causing intermittent gastric outlet obstruction [1-3,8-13].

In the present case, the lesion was revealed by symptoms of intermittent gastric outlet obstruction rather than being discovered incidentally. The patient experienced severe epigastric pain and early postprandial vomiting. Upper gastrointestinal endoscopy showed a large pedunculated polyp measuring approximately 4 cm and arising from the gastric body. The polyp intermittently prolapsed through the pylorus into the duodenal bulb and subsequently returned to the gastric cavity, explaining the intermittent nature of the obstructive symptoms.

Pyloroduodenal prolapse of a gastric polyp is an uncommon cause of gastric outlet obstruction. Kumar et al. reported four patients aged 58-76 years who presented with postprandial vomiting, epigastric discomfort, gastrointestinal bleeding, or iron-deficiency anemia. The lesions ranged from 2 to 9 cm, arose from different gastric sites, and included an adenoma, two hyperplastic polyps, and a leiomyoma. Treatment consisted of endoscopic polypectomy in three cases and surgical resection in one, with resolution of symptoms and anemia [8].

Subsequent individual case reports have described predominantly older patients presenting with postprandial vomiting, epigastric pain, early satiety, weight loss, abdominal fullness, or iron-deficiency anemia. Most lesions were large and pedunculated and arose from the antrum, prepyloric region, subcardial region, or lesser curvature [9-13].

Gencosmanoglu et al. reported a 62-year-old woman with intermittent nausea and vomiting caused by a 30-mm sessile hyperplastic prepyloric polyp partially obstructing the pylorus. The lesion was successfully removed by endoscopic polypectomy [9].

Freeman reported a 69-year-old man with iron-deficiency anemia and postprandial abdominal fullness. Endoscopy revealed a pedunculated antral adenomatous polyp prolapsing through the pylorus into the duodenum. Histological examination after endoscopic excision demonstrated high-grade dysplasia and a focus of intramucosal carcinoma without submucosal invasion [10].

Parikh et al. described an 89-year-old woman with early satiety, postprandial nausea, epigastric discomfort, weight loss, and iron-deficiency anemia. Endoscopy revealed a 20-mm pedunculated antral hyperplastic polyp prolapsing into the pylorus. Endoscopic polypectomy resulted in resolution of the obstructive symptoms [11].

Burud et al. reported a 61-year-old woman with epigastric pain and intermittent vomiting caused by a large pedunculated polyp arising along the lesser curvature and extending into the first portion of the duodenum. Endoscopic polypectomy revealed a hyperplastic polyp containing early gastric carcinoma. During two years of surveillance, mild-to-moderate dysplasia was subsequently detected at the previous resection site. Laparoscopic wedge resection was performed, and the surgical specimen showed a hyperplastic polyp with low-grade dysplasia [12].

Zorzetti et al. reported an 89-year-old woman with vomiting and mild epigastric pain. Endoscopy demonstrated a 4-cm pedunculated hyperplastic antral polyp prolapsing into the duodenal bulb and producing a ball-valve-type intermittent obstruction. The lesion was successfully resected endoscopically [13].

Most previously reported prolapsing polyps were hyperplastic or adenomatous lesions without deeply invasive malignancy and were successfully managed by endoscopic polypectomy alone. Nevertheless, the cases reported by Freeman and Burud demonstrate that dysplasia or early carcinoma may be present within a prolapsing gastric polyp [10,12].

Our case differs from most previous observations summarized in Table 1 because of the location of the lesion in the gastric body, its prolapse toward the duodenal bulb, and, most importantly, the presence of a moderately differentiated invasive adenocarcinoma arising in a tubular adenoma. The carcinoma invaded the submucosa to a depth of 2,400 µm. This depth of invasion represents deep submucosal invasion and placed the lesion outside the accepted criteria for curative endoscopic resection, thereby justifying additional gastrectomy with lymph node dissection.

**Table 1:** Published cases of gastric polyps prolapsing through the pylorus and causing gastric outlet obstruction.

Author and year	Patient	Clinical presentation	Location and size	Histological diagnosis	Treatment
Kumar et al., 1996 [8]	76-year-old woman	Recurrent epigastric pain, postprandial vomiting	Pedunculated polyp, 80 mm, subcardial, prolapsed through the pylorus	Adenoma without signs of malignancy	Endoscopic polypectomy
Gencosmanoglu et al., 2003 [9]	62-year-old woman	Intermittent nausea and vomiting	Sessile prepyloric polyp, 30 mm, partially obstructing the pylorus	Hyperplastic polyp without malignancy	Endoscopic polypectomy
Freeman, 2005 [10]	69-year-old man	Iron-deficiency anemia and postprandial abdominal fullness	Pedunculated antral polyp prolapsing through the pylorus into the duodenum; size not clearly reported	Adenomatous polyp with high-grade dysplasia and intramucosal carcinoma, without submucosal invasion	Endoscopic excision
Parikh et al., 2010 [11]	89-year-old woman	Early satiety, postprandial nausea, epigastric discomfort, weight loss, and iron-deficiency anemia	Pedunculated antral polyp, approximately 20 mm, prolapsing into the pylorus	Hyperplastic polyp without malignancy	Endoscopic polypectomy
Burud et al., 2018 [12]	61-year-old woman	Epigastric pain and intermittent vomiting	Large pedunculated polyp along the lesser curvature, extending into the first portion of the duodenum	Hyperplastic polyp containing early gastric carcinoma; subsequent dysplasia at the previous resection site	Endoscopic polypectomy, surveillance, then laparoscopic wedge resection
Zorzetti et al., 2021 [13]	89-year-old woman	Vomiting and mild epigastric pain	Pedunculated antral polyp, 40 mm, prolapsing into the duodenal bulb	Hyperplastic polyp without malignancy	Endoscopic resection
Present case, 2026	69-year-old woman	Intermittent gastric outlet obstruction with epigastric pain, early postprandial vomiting, and dyspepsia	Pedunculated gastric body polyp, approximately 40 mm, prolapsing toward the duodenal bulb	Moderately differentiated invasive adenocarcinoma arising in a tubular adenoma, with submucosal invasion to a depth of 2,400 µm	Endoscopic polypectomy followed by subtotal gastrectomy with lymph node dissection

Another important feature of the present case was the discrepancy between the suspicious endoscopic appearance and the initial histopathological findings. The initial forceps biopsies, obtained from the ulcerated apical area of the polyp, showed only acute inflammatory and ulcerative changes, without dysplasia or malignancy in the sampled tissue.

This discrepancy can be explained by surface ulceration, the superficial nature of forceps biopsies, and the heterogeneous distribution of dysplasia and invasive carcinoma within a large polypoid lesion [14]. Forceps biopsies sample only a limited part of the lesion and may therefore underestimate the grade of dysplasia or fail to detect a focal invasive component [14]. Thus, in the present case, the initially non-neoplastic biopsy findings were most likely attributable to sampling bias rather than to the absence of underlying neoplasia.

This phenomenon has been demonstrated in larger studies. In a series of 745 lesions initially diagnosed as low-grade gastric adenomas by forceps biopsy, the final resection specimen showed high-grade adenoma in 63 lesions and carcinoma in 49. The overall histological discrepancy rate was 15.4% after exclusion of non-neoplastic lesions. Depressed morphology, erythema, and a lesion size greater than 1.5 cm were independently associated with histological discrepancy [14].

Therefore, a negative or non-neoplastic forceps biopsy should not be considered sufficient to exclude neoplasia when there is a marked discrepancy between the biopsy result and the clinical or endoscopic appearance. This is especially important in large, symptomatic, ulcerated, prolapsing, erythematous, or morphologically suspicious lesions. Complete endoscopic resection, when technically feasible and considered safe, allows a substantially more reliable histological assessment than superficial forceps biopsies alone [14].

In our patient, endoscopic polypectomy played both a diagnostic and an initial therapeutic role. Examination of the complete specimen revealed a tubular adenoma containing low-grade dysplasia in approximately 20% of the lesion and high-grade dysplasia in approximately 80%, with an associated moderately differentiated invasive adenocarcinoma. The carcinoma extended beyond the muscularis mucosae and invaded the submucosa to a maximum depth of 2,400 µm.

The deep resection margin was free of carcinoma, with a clearance of 3 mm. No lymphovascular invasion or perineural invasion was identified. Tumor budding was also absent. Although tumor budding may be recorded as an additional histopathological feature, it is not included among the formal gastric criteria defining endoscopic curability in the European Society of Gastrointestinal Endoscopy (ESGE) recommendations; the ESGE budding criterion specifically applies to colorectal lesions [15].

The central clinical question was therefore whether the endoscopic resection could be considered curative. Assessment of curability after endoscopic resection of superficial gastric adenocarcinoma is based on the completeness and en bloc nature of resection, horizontal and vertical margin status, histological differentiation, tumor size, ulceration, lymphovascular invasion, and depth of submucosal invasion [15-18].

Histological differentiation is particularly relevant when assessing endoscopic curability. Well- and moderately differentiated adenocarcinomas are generally considered differentiated-type tumors and may meet curative endoscopic resection criteria across a broader range of clinicopathological settings. In contrast, poorly differentiated or other undifferentiated-type carcinomas are subject to more restrictive curability criteria because of their higher risk of lymph node metastasis [15-18]. Therefore, the degree of differentiation must be considered together with invasion depth, lymphovascular invasion, margin status, tumor size, and ulceration when determining the need for additional surgical treatment.

Current European recommendations define a gastric resection as high risk or non-curative when there is a positive vertical margin, lymphovascular invasion, poorly differentiated histology under specified conditions, or deep submucosal invasion exceeding 500 µm below the muscularis mucosae. Complete staging and strong consideration of additional surgical treatment are recommended after multidisciplinary discussion [15,16].

The updated French intergroup recommendations and the Japanese gastric cancer treatment guidelines similarly emphasize that endoscopic treatment is appropriate only for selected early gastric cancers with a negligible risk of lymph node metastasis. When the criteria for curative endoscopic resection are not met, additional gastrectomy with lymph node dissection should be considered according to the patient’s operative fitness and the estimated nodal risk [17,18].

In the present case, several findings were favorable: the lesion was completely retrieved, the deep margin was clear, the carcinoma was moderately differentiated and therefore belonged to the differentiated histologic category, and no lymphovascular or perineural invasion was identified. Nevertheless, the depth of submucosal invasion reached 2,400 µm, markedly exceeding the 500-µm threshold for superficial submucosal invasion. The resection was therefore non-curative because of deep submucosal invasion, irrespective of the clear deep margin.

Following multidisciplinary discussion, the patient underwent a four-fifths subtotal gastrectomy with regional lymph node dissection and transmesocolic gastrojejunal anastomosis. Histopathological examination of the surgical specimen revealed no residual carcinoma. The proximal and distal surgical margins were tumor-free, and all 13 examined lymph nodes were negative for metastatic involvement.

The absence of residual tumor and lymph node metastasis does not invalidate the indication for additional surgery [15-18]. The objective of gastrectomy following a non-curative endoscopic resection is not limited to removing possible residual local disease. It also allows assessment and treatment of occult lymph node metastasis, which cannot be reliably excluded by a clear endoscopic resection margin or by preoperative cross-sectional imaging alone [15-18].

The non-neoplastic gastric mucosa showed mild follicular, mildly active, non-atrophic chronic gastritis with areas of complete intestinal metaplasia, without dysplasia or H. pylori infection. Complete and focal intestinal metaplasia generally carries a lower risk than incomplete or extensive intestinal metaplasia [16,19]. However, the interpretation of this finding must consider the patient’s personal history of gastric adenocarcinoma and the condition of the remaining gastric mucosa.

The Management of Epithelial Precancerous Conditions and Lesions in the Stomach III (MAPS III) guidelines recommend high-quality endoscopic assessment with virtual chromoendoscopy for the diagnosis and staging of gastric precancerous conditions. Surveillance is recommended for extensive intestinal metaplasia, advanced Operative Link on Gastritis Assessment (OLGA) or Operative Link on Gastric Intestinal Metaplasia Assessment (OLGIM) stages, or additional risk factors. Conversely, systematic surveillance is not generally recommended for mild-to-moderate intestinal metaplasia restricted to a limited gastric region in the absence of other risk factors [16].

The American Gastroenterological Association (AGA) similarly recommends testing for and eradicating H. pylori in patients with gastric intestinal metaplasia but does not recommend routine surveillance for every patient. Surveillance may be considered following individualized risk assessment and shared decision-making in patients with higher-risk features [19].

In the present patient, surveillance should not be justified solely by the focal complete intestinal metaplasia. Nevertheless, individualized endoscopic surveillance of the gastric remnant may reasonably be considered because of the previous invasive gastric adenocarcinoma, the adenomatous pathway of carcinogenesis, and the potential risk of metachronous gastric neoplasia. The surveillance strategy should be determined during multidisciplinary follow-up.

Overall, this case illustrates a rare presentation of moderately differentiated invasive gastric adenocarcinoma arising in a large pedunculated tubular adenoma and revealed by intermittent gastric outlet obstruction secondary to pyloroduodenal prolapse. It highlights three main clinical messages: first, large gastric adenomas may harbor invasive carcinoma; second, superficial biopsies may fail to detect dysplasia or malignancy in large and histologically heterogeneous polypoid lesions; and third, additional surgery should be considered after a non-curative endoscopic resection when deep submucosal invasion creates a clinically relevant risk of occult lymph node metastasis.

## Conclusions

Pyloroduodenal prolapse of a pedunculated gastric polyp is an exceptional cause of intermittent gastric outlet obstruction. This case highlights the importance of considering this diagnosis in patients presenting with recurrent postprandial vomiting and a large mobile gastric lesion. Careful endoscopic assessment and complete histopathological examination are essential to guide subsequent management and ensure an appropriate oncological approach.
